# Smoking status impacts microRNA mediated prognosis and lung adenocarcinoma biology

**DOI:** 10.1186/1471-2407-14-778

**Published:** 2014-10-24

**Authors:** Emily A Vucic, Kelsie L Thu, Larissa A Pikor, Katey SS Enfield, John Yee, John C English, Calum E MacAulay, Stephen Lam, Igor Jurisica, Wan L Lam

**Affiliations:** Department of Integrative Oncology, British Columbia Cancer Research Centre, Vancouver, British Columbia V5Z 1L3 Canada; Department of Surgery, Vancouver General Hospital, Vancouver, British Columbia Canada; Department of Pathology, Vancouver General Hospital, Vancouver, British Columbia Canada; University Health Network Division of Cancer Informatics, Ontario Cancer Institute and Princess Margaret Hospital, Toronto, Ontario Canada; Department of Computer Science, University of Toronto, Toronto, Ontario Canada

**Keywords:** Lung adenocarcinoma, miRNA, Current smoker, Former smoker, Never smoker, Reversible, Survival, Smoking specific

## Abstract

**Background:**

Cigarette smoke is associated with the majority of lung cancers: however, 25% of lung cancer patients are non-smokers, and half of all newly diagnosed lung cancer patients are former smokers. Lung tumors exhibit distinct epidemiological, clinical, pathological, and molecular features depending on smoking status, suggesting divergent mechanisms underlie tumorigenesis in smokers and non-smokers. MicroRNAs (miRNAs) are integral contributors to tumorigenesis and mediate biological responses to smoking. Based on the hypothesis that smoking-specific miRNA differences in lung adenocarcinomas reflect distinct tumorigenic processes selected by different smoking and non-smoking environments, we investigated the contribution of miRNA disruption to lung tumor biology and patient outcome in the context of smoking status.

**Methods:**

We applied a whole transcriptome sequencing based approach to interrogate miRNA levels in 94 patient-matched lung adenocarcinoma and non-malignant lung parenchymal tissue pairs from current, former and never smokers.

**Results:**

We discovered novel and distinct smoking status-specific patterns of miRNA and miRNA-mediated gene networks, and identified miRNAs that were prognostically significant in a smoking dependent manner.

**Conclusions:**

We conclude that miRNAs disrupted in a smoking status-dependent manner affect distinct cellular pathways and differentially influence lung cancer patient prognosis in current, former and never smokers. Our findings may represent promising biologically relevant markers for lung cancer prognosis or therapeutic intervention.

**Electronic supplementary material:**

The online version of this article (doi:10.1186/1471-2407-14-778) contains supplementary material, which is available to authorized users.

## Background

Lung cancer is the most common cause of cancer-related death [1]. Cigarette smoke is a significant contributor to morbidity and mortality worldwide, and is the number one risk factor for lung cancer. However, half of newly diagnosed patients are former smokers (FS) and up to 25% of patients are never smokers (NS) [2, 3]. Clinically, smoking history can be used to subclassify histologically similar tumors based on smoking status-associated molecular and clinical features, and can inform therapeutic decisions [3–7]. With the success of smoking cessation programs, smoking incidence continues to drop in developed countries and an increasing proportion of FS and NS patients are being diagnosed in the clinic. Consequently, there is an urgent need for an improved understanding of the molecular mechanisms underlying lung cancer biology in patients with different smoking histories.

MicroRNAs (miRNAs) negatively regulate mRNA expression through direct inhibition of translation or induction of mRNA degradation [8]. They are key contributors to smoking response, tumorigenesis, progression, and treatment response, and therefore represent promising and biologically relevant biomarkers [9–15]. We hypothesized that, analogous to distinct smoking status-related patterns of DNA and mRNA alterations, miRNAs display smoking status-specific patterns of disruption in both non-malignant and malignant lung tissues from lung cancer patients. We investigated the effects of smoking on the miRNA transcriptome of lung tumors and parenchymal tissues from current smokers (CS), FS and NS. miRNA-mRNA gene networks were built to determine the potential biological consequences associated with miRNA disruption in these three groups, and we evaluated the potential clinical significance of our findings in relation to patient survival in the context of smoking status.

## Methods

### Samples

Fresh-frozen lung adenocarcinoma (LUAC) tumor and patient-matched non-malignant lung parenchymal tissue was collected for 94 treatment naïve patients at Vancouver General Hospital under informed, written patient consent and with approval from the University of British Columbia-BC Cancer Agency Research Ethics Board (Table 1). Non-malignant samples were collected from areas >2 cm away from the tumor. Tissue microdissection was guided by a lung pathologist to ensure >80% tumor cell or >80% non-malignant cell content. Total RNA was extracted using Trizol reagent. For this study, we used the following definitions to define smoking status: NS are patients who smoked fewer than 100 cigarettes in their lifetime; CS are patients who were smoking at the time of diagnosis; and FS are individuals who had stopped smoking at least one year prior to diagnosis.

**Table 1:** **Clinical information for lung adenocarcinoma samples profiled**

Characteristic	NS ^1^	CS ^2^	FS ^3^
**Number**	27	43	24
**Sex**			
Male	7 (26%)	13 (30%)	9 (38%)
Female	20 (74%)	30 (70%)	15 (62%)
**Average age**	71	64	71
**Stage**			
I	16 (59%)	26 (60%)	16 (67%)
II	6 (22%)	11 (26%)	6 (25%)
III	5 (19%)	4 (9%)	1 (4%)
IV	0	2 (5%)	1 (4%)
**Ethnicity**			
Caucasian	8 (30%)	11 (26%)	1 (4%)
Asian	16 (59%)	0 (0%)	0 (0%)
Unknown	3 (11%)	32 (74%)	23 (96%)
**Average pack years**	0	46	47
**Average years quit**	n/a	< 1	15

^1^NS as patients who smoked fewer than 100 cigarettes in their lifetime. ^2^CS were defined as smoking at the time of diagnosis. ^3^FS as individuals who had stopped smoking at least one year prior to diagnosis.

### MiRNA sequencing

MiRNA-seq transcriptome profiles were generated using 1 μg of total RNA for each sample and were sequenced on the Illumina HiSeq 2000 platform as previously described [16]. Raw miRNA sequence libraries and sample information have been deposited in the NCBI Gene Expression Omnibus (GSE62182) ( http://www.ncbi.nlm.nih.gov/geo/). Reads were aligned to NCBI GRCh37 reference genome and miRBase v18 using the BWA algorithm [17], and multiple alignment locations resolved as previously described [16]. Full description of library construction, sequencing, read pre-processing, alignment and annotation are previously described [16]. MiRNA expression was quantified as reads per kilobase per million (RPKM). In total, 1372 unique miRNAs were detected across 188 libraries. MiRNAs with RPKMs <1 were considered not expressed, and miRNAs with no detectable expression across the entire cohort of tumor or non-malignant samples were disregarded, resulting in 927 miRNAs for subsequent statistical analyses.

### The Cancer Genome Atlas (TCGA) cohort

MiRNA sequencing data for lung adenocarcinoma (LUAC) tumors were obtained from the TCGA for use as an external cohort for validation purposes as well as for combining with our own dataset to perform miRNA survival association analyses. Expression profiles from the TCGA were processed as described for ‘Level 3 data’ in the TCGA data compendium (2011 Cancer Genome Atlas Network). Detailed descriptions of the use of TCGA data are described below.

### Statistical analyses

#### Unsupervised hierarchical clustering of miRNA expression profiles

Unsupervised hierarchical clustering using Ward’s method was performed on all samples (n = 188), tumor samples only (n = 94), and non-malignant samples only (n = 94) using *Partek Genomics Suite* software. A Fisher’s Exact test and Chi-square tests were performed to assess the distribution of tumor and non-malignant profiles, and distribution of the three smoking types within the identified clusters, respectively. A Student’s t-test was used to assess differences in pack years and years quit for CS and FS. A multivariate analysis of variance (MANOVA) test was performed to determine which clinical factors were most strongly associated with grouping of non-malignant and tumor miRNA expression profiles into distinct clusters. A Kruskal-Wallis (KW) test was performed to identify miRNA differentially expressed between the clusters identified for both tumor and non-malignant tissues. For all statistical tests, a p-value <0.05 was considered significant.

#### MiRNAs modulated in response to smoking

To identify miRNAs whose expression is likely modulated in response to smoking, we performed a non-parametric permutation test using 10,000 permutations, between non-malignant CS and NS tissues (CSN and NSN, respectively). Permutation scores were corrected for multiple testing using the Benjamini and Hochberg (B-H) method. miRNAs that had a B-H corrected p <0.05 and an average fold change >2.0 or <0.5 were considered differentially expressed (DE) between CSN and NSN tissues. To identify miRNAs recurrently, aberrantly expressed in lung tumors of each smoking group (i.e., CS, FS, and NS), we applied the following criteria: i) pairwise Wilcoxon Sign Rank test B-H multiple testing corrected p <0.05, and ii) tumor/normal fold change >2 (overexpression) or <0.5 (underexpression) in at least 25% of the tumors for that particular smoking group. MiRNAs satisfying these criteria in only one group, were considered smoking status-specific and further investigated in the TCGA cohort. Tumor tissues from 80 FS, 42 CS, and 16 NS were used to investigate the reproducibility of miRNAs we identified as disrupted in a smoking status-specific manner. Low numbers of non-malignant lung parenchymal tissues at the time of writing for the various smoking groups (12 FS, 9 CS and 2 NS) precluded us from validating our non-malignant tissue findings and required us to use pooled non-malignant samples of matched smoking history to calculate miRNA fold change for each TCGA tumor. Therefore, miRNAs were considered validated if the frequency of over- or underexpression was found to significantly differ between groups (Fisher’s exact test, p <0.05) and there was a minimum disruption frequency difference of 15% between groups in the TCGA cohort.

#### Generation of predicted miRNA-transcript interaction networks

MiRNAs identified as preferentially disrupted in one smoking status group were inputted into the *microRNA Data Integration Portal ver 2* (*miRDIP*; http://ophid.utoronto.ca/mirDIP), which integrates 13 microRNA target prediction algorithms and six microRNA prediction databases to predict miRNA-transcript (mRNA) interactions [18]. For this study, we used stringent miRNA target prediction criteria by considering only predictions that were supported by at least six sources. Interactions between miRNAs and their predicted mRNA targets were then visualized as networks using *NAViGaTOR v2.14* ( http://ophid.utoronto.ca/navigator) [19, 20]. Two interaction networks were generated: 1) a network based on miRNAs specifically deregulated in one smoking group using all significant gene targets identified by miRDIP, and 2) a network based on miRNAs specifically disrupted in CS, FS, or NS and miRNAs commonly disrupted between all groups using only significant gene targets identified by miRDIP that are known to be associated with lung cancer patient survival [21]. Only the most highly connected miRNAs were used to build and visualize the networks. Pathway analysis was performed on biologically validated mRNA targets (miRTarBase v3.5) of miRNAs disrupted in a smoking status-specific manner using Ingenuity Pathway Analysis.

#### MiRNA survival associations in lung cancer cohorts

Associations between miRNA expression and patient survival were assessed using a log rank, Mantel-Haenszel test, with a p <0.05 considered significant. Patients were divided into tertiles based on miRNA expression, and survival for patients in the top and bottom tertiles was compared. Only miRNAs detectably expressed in at least two thirds of patients were assessed to ensure adequate separation between high and low expressing groups for statistical analysis. To enable assessment of smoking status-specific survival associations, we combined miRNA expression and outcome data for our own patient cohort (n = 91; 22 FS, 42 CS, 27 NS) and the TCGA LUAC cohort (n = 127; 80 FS, 33 CS, and 14 NS). In total, the combined cohort contained 218 patients including 102 FS, 75 CS, and 41 NS. Survival analyses were performed on all patients and each specific smoking group. Kaplan-Meier plots were generated using GraphPad Prism 6 software. Cox proportional hazards (COXPH) multivariate survival analyses were also performed to determine the influence of miRNA expression and other clinical covariates (age, gender, ethnicity, tumor stage, and smoking status) on patient outcome considering all LUAC patients for the combined cohort (n = 218). A COXPH p <0.1 was considered significant. A multivariate analysis of variance (MANOVA) was also performed on the entire LUAC cohort to determine whether miRNA expression was significantly associated with any of these clinical covariates. A MANOVA p-value <0.05 was considered significant. Expression data for 38 CS LUAC profiles used to calculate the association of EZH2 expression with patient survival was acquired from the Early Detection Research Network (EDRN, http://edrn.nci.nih.gov/science-data), and processed as previously described [7, 22]. EZH2 survival analysis was performed as described above.

## Results

### MiRNA expression profiles cluster based on malignancy and smoking histories

**Figure 1:**
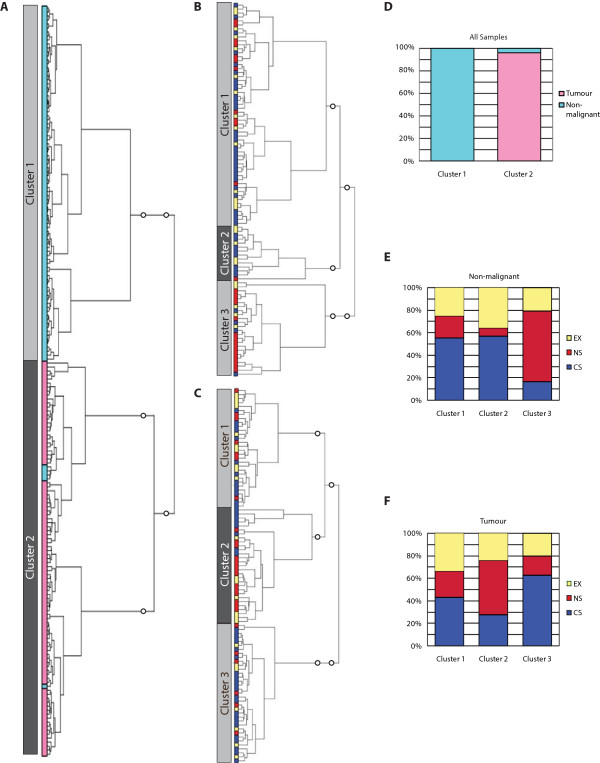
**Unsupervised hierarchical clustering of lung tumor and non-malignant miRNA expression profiles.** Clustering of all 188 miRNA expression profiles revealed two distinct clusters, one comprised of non-malignant samples (teal), and the other comprised of mostly tumors (pink) **(A)**. The clusters identified were associated with malignancy, as clusters 1 and 2 were significantly enriched for non-malignant and tumor profiles, respectively (Fisher’s Exact test p =2.2 × 10^-16^) Clustering of non-malignant tissues only **(B)** and tumors only **(C)** revealed three clusters. **(D)**. Assessment of the distribution of CS, FS, and NS within the clusters identified in non-malignant samples revealed enrichment for CS and FS in clusters 1 and 2 compared to cluster 3 (Chi-square test p =5.0 × 10^-4^) **(E)**. The same trend was observed in the clusters identified based on tumor profiles (Chi-square test p =0.023) **(F)**.

To determine whether miRNA expression was associated with smoking status in non-malignant and lung tumor tissues, we performed unsupervised hierarchical clustering on 927 miRNAs with detectable expression across the 188 lung tumor and non-malignant tissues. Clustering revealed miRNA expression segregated samples based on malignancy and smoking status (Figure 1). When all profiles were considered, tumor and non-malignant samples clustered separately, with a significant difference between the two clusters (Figure 1A and D, Fisher’s Exact test, p =2.2 × 10^-16^). Clustering of non-malignant profiles revealed three clusters that were significantly different in smoking status composition (Figure 1B and E, Chi-square test, p =5.0 × 10^-4^). A similar clustering pattern was observed for tumor profiles (Figure 1C and F, Chi-square test, p =0.023). Assessment of the miRNAs differentially expressed between the identified clusters revealed that similar miRNAs contribute to cluster grouping in both tumor and non-malignant tissue. For example, 57% of miRNAs that were significantly differentially expressed across the three tumor clusters were also differentially expressed across the three non-malignant clusters, while 36% of miRNAs significantly differentially expressed between the non-malignant clusters were also differentially expressed across the tumor clusters (Additional file 1). Multivariate analysis revealed smoking to be the clinical variable most strongly associated with cluster grouping in non-malignant tissue (F-value =10.05, p =2.3 × 10^-3^), whereas in tumors, age and years quit were the most significant variables associated with clustering (F-value =3.13, p =0.015 and F-value =15.80, p =0.038, respectively) (Additional file 2). We also observed a significant difference in pack years for FS tumors among the two clusters dominated by CS and FS tumors (Student’s t-test, p =0.030); however, pack years was not a significant factor between clusters of non-malignant tissues or between clusters dominated by CS tumors. Collectively, these results suggest miRNA expression profiles in both tumor and non-malignant lung tissues are dependent on smoking histories, but that heterogeneity within ever-smoking groups (i.e. CS and FS) exists.

### MiRNAs are differentially expressed between non-malignant lung tissues of CS and NS with lung cancer

Based on the observed clustering patterns, we aimed to identify miRNAs differentially expressed in non-malignant tissues of CS (CSN) and NS (NSN), as these two groups represent the most extreme smoking phenotypes. 37 miRNAs were significantly differentially disrupted between CSN and NSN; 25 of which were overexpressed and 12 that were underexpressed in CSN relative to NSN (Additional file 3). Several of these miRNAs have been previously implicated in lung cancer including miR-106a, miR-107, miR-136, miR-142, miR-19a, miR-212, miR-339, miR-34b, miR-34c, and miR-449a.

### MiRNA expression in non-malignant tissue can be irreversibly altered in FS

Protein coding genes deregulated in response to active smoking display either reversible or irreversible expression upon smoking cessation [23–25]. Genes upregulated in response to smoking that remain overexpressed in lung tissues of CS and FS with lung cancer may indicate those smoking-related events involved in lung tumor development. We investigated this phenomenon with respect to miRNA expression in FS non-malignant tissue (FSN), and identified two miRNAs exhibiting patterns consistent with reversible expression and 15 with irreversible expression in FSN (Additional file 4). Interestingly, the majority of these miRNAs have been associated with cancer, and four specifically in lung cancer (miR-107, 142, 339, and 34c).

### MiRNAs are recurrently altered in tumors from CS, FS and NS patients

To identify miRNAs recurrently differentially expressed in tumors from each smoking group, we compared expression profiles for tumor and patient-matched non-malignant lung tissues of CS, FS and NS. This analysis revealed 232 overexpressed and 58 underexpressed miRNAs in current smoker tumors (CST); 257 overexpressed and 47 underexpressed miRNAs in former smoker tumors (FST); and 263 overexpressed and 41 underexpressed miRNAs in never smoker tumors (NST) (Additional file 5). Overall, the majority of miRNAs were overexpressed (304/366, 83%). 65% (196/304) of overexpressed miRNAs and 58% (36/62) of underexpressed miRNAs were shared between CS, FS and NS tumors. Many of these (miR-17, miR-21, miR-106a let-7a, let-7c, miR-101, and miR-143, for example) are well known lung cancer miRNAs (Additional file 6). The identification of shared patterns of miRNA deregulation within CS, FS and NS lung tumors suggests that miRNAs likely participate in common mechanisms of tumorigenesis in lung adenocarcinoma (LUAC).

66 miRNAs were frequently altered in only one tumor group: 25 in CS (14 overexpressed and 11 underexpressed), 14 in FS (12 overexpressed and 2 underexpressed), and 27 in NS (26 overexpressed and 1 underexpressed) (Additional file 7). We refer to these miRNAs, including those preferentially disrupted in NS, as smoking status-specific. In addition to recurrent expression deregulation in tumor relative to non-malignant tissue, miRNAs affected by smoke exposure could be expected to exhibit differential expression between CS and NS tumor tissues. Of the 25 CS-specific and 27 NS-specific miRNAs, 8/25 and 4/27 also showed differential expression when CS and NS tumors were compared (irrespective of status in non-malignant tissues) (Additional file 7). The established cancer-related functions of some of the smoking status-specific miRNAs, such as overexpression of miR-7, miR-27a, miR-93, miR-372, and underexpression of miR-138, miR-381, miR-582 [26–33], suggest miRNAs are also likely involved in promoting tumorigenesis in a smoking status-dependent manner.

To assess the robustness and reproducibility of our findings we analyzed expression of our smoking status-specific miRNAs in an external cohort. Of the 66 miRNAs altered in a smoking status-specific manner, 57 were annotated in the LUAC dataset from The Cancer Genome Atlas (TCGA); however, 12 of these 57 miRNAs were not detectably expressed in the smoking group of interest. Therefore, 45 miRNAs were amenable to validation testing. In addition to a low number of patient-matched tumor and non-malignant tissues pairs (n = 23), inspection of the TCGA data revealed lower overall RPKM (Reads Per Kilobase of transcript per Million mapped reads) counts for most miRNAs detected in comparison with our own dataset (Additional file 8). Thus, we had to apply a different analysis strategy for validation (see Methods), resulting in 4 of the 45 assessable miRNAs validating as altered in a smoking status-specific manner: miR-129 overexpressed in CS, miR-152 overexpressed in NS, miR-3065 underexpressed in CS, and miR-511 underexpressed in CS (Additional file 9).

### Disrupted miRNA networks in tumors indicate selection of smoking status-specific target genes

**Figure 2:**
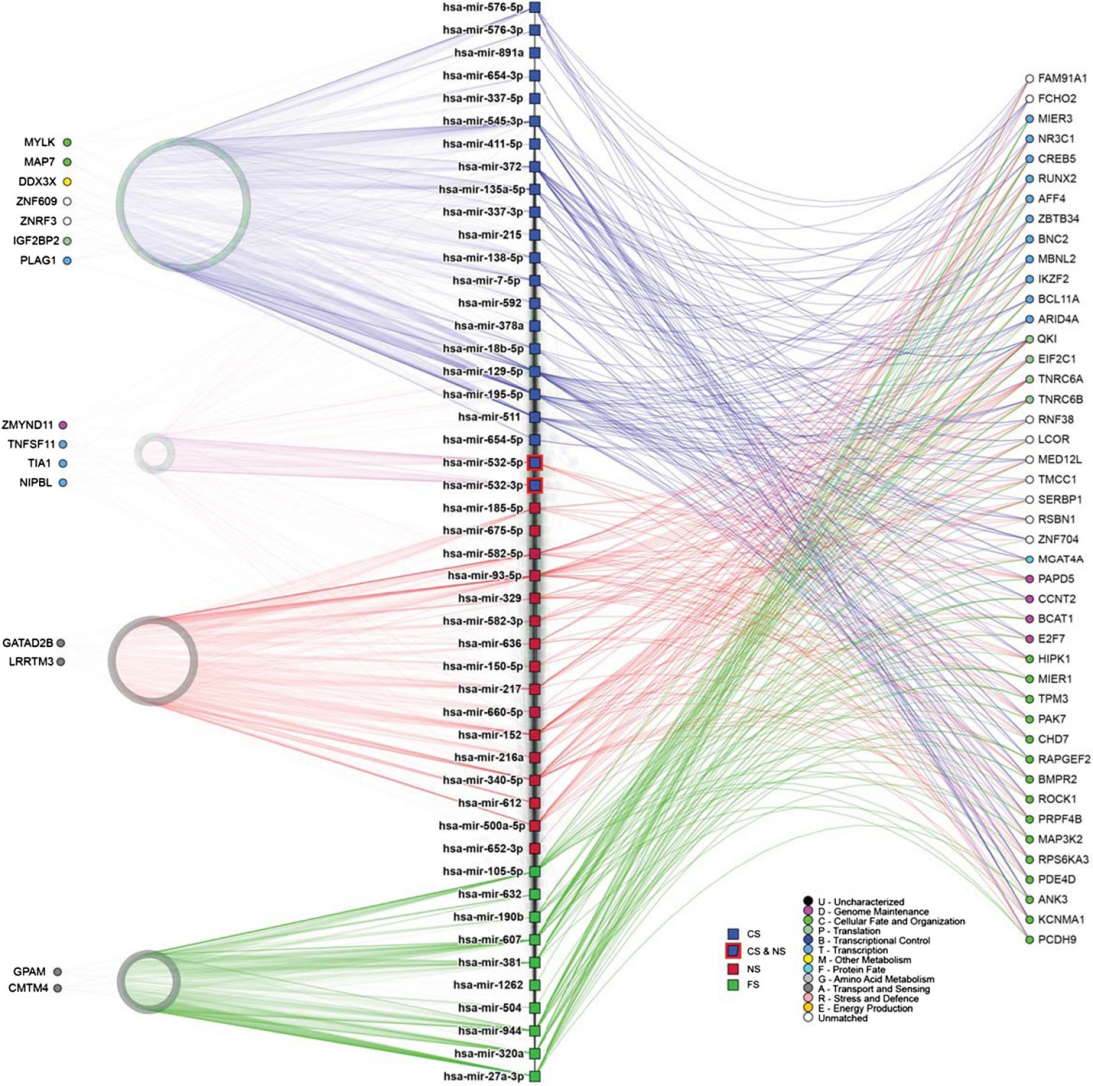
**Network interactions between deregulated lung tumor miRNAs and their predicted mRNA targets.** MiRNAs specifically disrupted in CST, FST, or NST tumors were inputted into mirDIP to identify their predicted gene targets (i.e., mRNA transcripts predicted by at least six miRNA target prediction algorithms). The network of identified miRNA-mRNA interactions was then generated and visualized using NAViGaTOR. Only the most highly connected miRNAs were used to build the network. miRNAs specifically deregulated in CST, FST, and NST are indicated by blue, green, and red colored square nodes, respectively. Predicted mRNA targets are represented as circular nodes. Edges indicate miRNA-mRNA interactions, and are color-coded to match smoking group specificity of miRNA deregulation. Numerous target genes were shared by miRNAs specifically deregulated in CS, FS, and NS, as shown to the right of the miRNAs list in the centre. Conversely, targets unique to specific smoking groups are indicated to the left of the list. Predicted targets uniquely mapping to miRNAs disrupted in one smoking status group are represented by circles on the left; 1,162 mRNA targets were unique to miRNAs altered in CST, 927 to NST miRNAs, and 770 to FST miRNAs. Conversely, 1,399 mRNA-miRNA targets were shared between miRNAs altered uniquely in each smoking status group. MiR-532 was underexpressed in CST and overexpressed in NST. Gene Ontology terms associated with predicted target genes are indicated by target gene shading.

To elucidate signaling pathways and biological processes disrupted by smoking status-specific miRNAs, mRNA target genes were identified using miRDIP (microRNA Data Integration Portal) with stringent filters (i.e. prediction by at least 6 different algorithms). Smoking status-specific miRNAs were predicted to affect a large number of unique mRNA targets in CS (n = 1,162 genes), FS (n = 770 genes), and NS (n = 927 genes), which could indicate that distinct cellular pathway selection occurs in different smoking and non-smoking environments (Figure 2, left side). Conversely, common mRNA target genes (n = 1,399) (Figure 2, right side) may indicate selection of genes deregulated in LUAC in general. While the number of connections (i.e. network edges) between miRNA and mRNA targets did not differ between CS, FS, and NS tumor groups, CS tumor (CST)-specific miRNAs targeted mRNAs with numerous Gene Ontology functions, including cellular fate and organization, metabolism, genome maintenance, transcription, and translation, whereas in FS tumor (FST)- and NS tumor (NST), mRNA targets largely corresponded to similar functions, including transport and sensing (Figure 2). As an independent method of assessing the potential biological implications of miRNA disruption, we performed pathway analysis on biologically validated targets of miRNAs (as annotated in miRTarBase v3.5) specifically deregulated in one smoking group. We found not only expected commonalities in known cancer pathways across all groups, but also biological pathways that were uniquely disrupted in specific smoking groups; for example, SAPK/JNK signaling in NS and ERK5 in CS (Additional files 10 and 11).

### Lung cancer prognostic genes are targeted by miRNAs disrupted in a smoking status-specific manner

**Figure 3:**
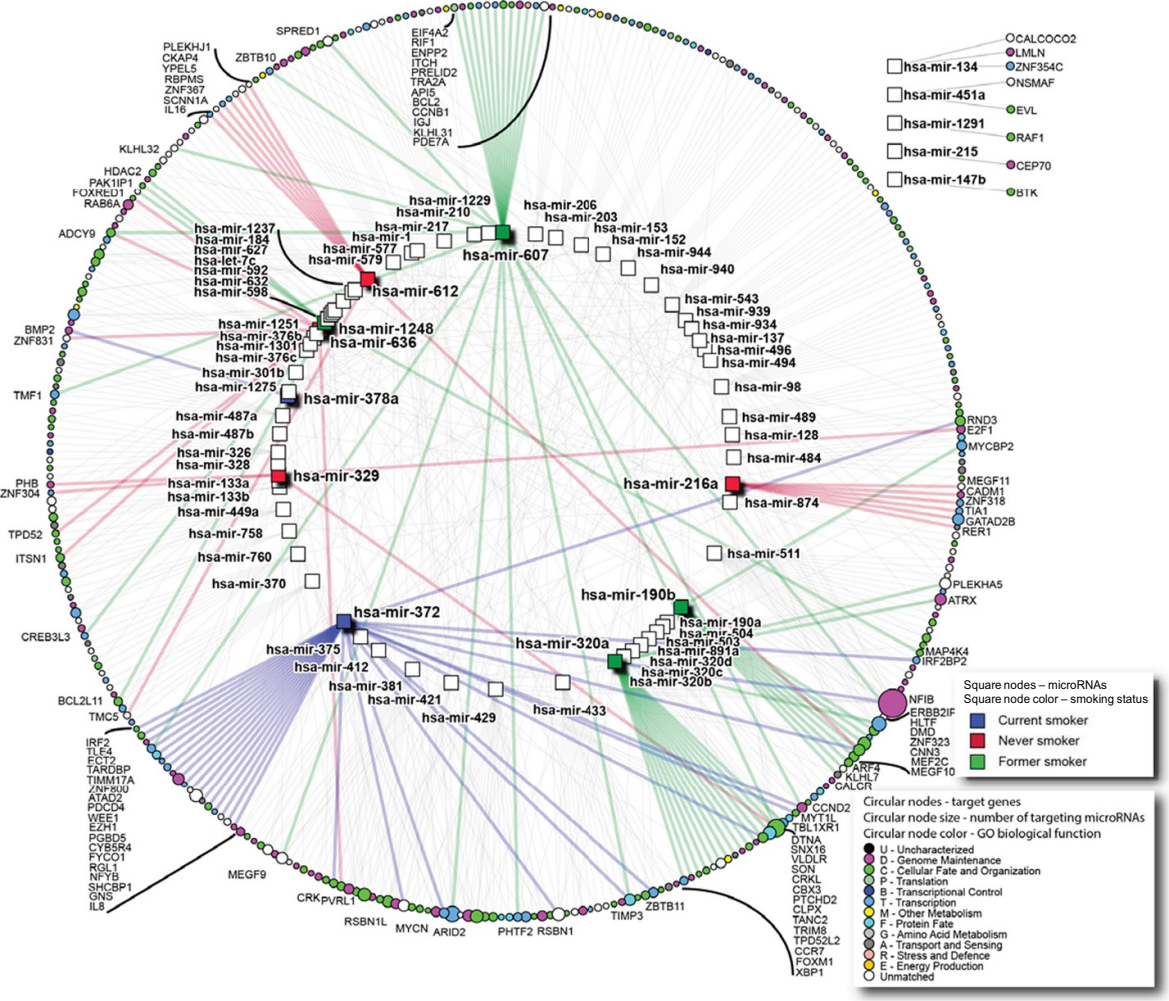
**Predicted interaction between prognostic lung cancer genes and deregulated lung tumor miRNAs.** MiRNAs specifically disrupted in CS, FS, or NS tumors as well as miRNAs frequently disrupted across the groups were inputted into mirDIP to identify their predicted gene targets. The network of identified miRNA-mRNA interactions was then generated and visualized using NAViGaTOR, but was restricted to predicted target genes that are known to have prognostic significance in lung cancer. MiRNAs specifically deregulated in a single smoking group are indicated by colored square nodes. MiRNAs disrupted in multiple groups are indicated by white square nodes. Connections for miRNAs commonly disrupted among the smoking groups are indicated by grey edges, while blue, green and red edges indicate miRNA-mRNA interactions specific to CST, FST, and NST, respectively. Predicted targets are depicted as circular nodes, with shading corresponding to Gene Ontology terms associated with gene function. The degree of connectivity for gene targets is depicted by the target node size, where larger circular nodes indicate genes targeted by a greater number of different miRNAs. In total, the network is comprised of 75 miRNAs and 385 prognostic target genes. Most miRNAs are well connected to prognostic genes, with more connections for CST- and FST-specific miRNAs and fewer connections for NST-specific miRNAs. MiR-372, miR-607, and miR-543 were among the miRNAs most highly connected to lung cancer prognostic gene targets.

To assess the potential prognostic implications of miRNA deregulation in lung cancer, we used a curated list of 1,066 lung cancer prognostic genes compiled by Zhu *et al.* [21] to build a miRNA-transcript interaction network comprised of both smoking status-specific miRNAs (n = 66, Figure 3, colored square nodes) and miRNAs frequently altered across all LUAC groups (n = 232 Figure 3, white square nodes). Of the 1,066 prognostic genes, 358 (34%) were predicted targets of the most highly connected miRNAs (n = 75) used to derive the network (Additional file 12). Interestingly, the majority of miRNAs were highly connected to the same lung cancer prognostic genes, and vice versa. For instance, miR-372, a miRNA identified as specifically overexpressed in CST, was connected to 31 different lung cancer prognostic genes. Conversely, *nuclear factor I/B* (*NFIB*), a critical regulator of fetal lung maturation and lung mesenchymal and epithelial cell proliferation [34, 35], was identified as a target of 16 unique miRNAs, highlighting the potential importance of this gene in LUAC. Moreover, of the miRNAs disrupted in a smoking status-specific manner, CS (blue) and FS (green) miRNAs demonstrated a higher number of connections to lung cancer prognostic genes than NS-specific miRNAs (red). Taken together, the miRNAs targeting prognostic genes and the smoking specificity we observed, emphasizes not only the biological relevance of miRNA disruption but also the potential clinical implications of these miRNA.

### MiRNAs disrupted specifically in CS, FS, or NS tumors are associated with lung cancer patient outcome

Due to the scarcity of publically available cohorts large enough to enable statistical analyses of survival for patients with different smoking histories, analysis of miRNA expression in relation to CS, FS or NS lung cancer patient survival has, to our knowledge, not been previously assessed. By combining miRNA expression and patient survival data from our LUAC cohort (n = 91) with the TCGA LUAC cohort (n = 127; combined cohort n = 218 with 102 FST, 75 CST and 41 NST), we addressed these challenges and performed the first smoking status-specific miRNA survival analysis of LUAC.

**Figure 4:**
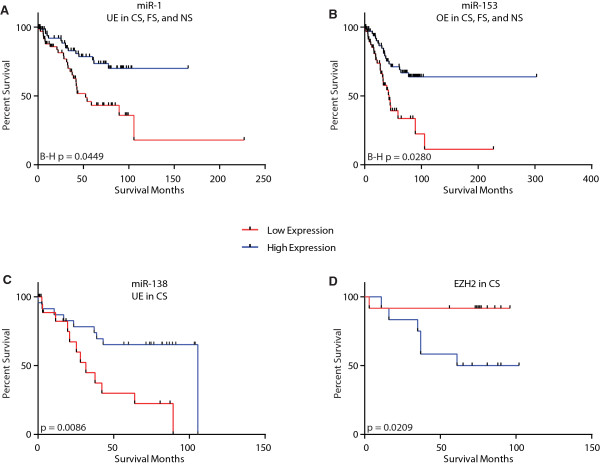
**MiRNAs deregulated in CS, FS and NS lung adenocarcinomas are associated with patient survival.** Associations between miRNA expression and LUAC patient survival were assessed for miRNAs identified as deregulated in LUAC using a logrank, Mantel-Haenszel test. Survival analyses were performed independently for tumors from all smoking status groups, CS, FS, and NS patients. Numerous miRNAs commonly disrupted across CS, FS, and NS were significantly associated with LUAC patient survival, including miR-1 **(A)** and miR-153 **(B)** (B-H p <0.05) which were both connected to multiple lung cancer prognostic genes from Figure 3. MiR-138, which was preferentially underexpressed in CS tumors, was also significantly associated with CS LUAC patient outcome, with low expression associated with poor survival **(C)** (p <0.05). High expression of EZH2, a biologically validated target of miR-138, showed a significant association with poorer survival in CS LUAC patients **(D)** (p <0.05). OE, overexpressed. UE, underexpressed. NS, never smokers. CS, current smokers. FS, former smokers. B-H, Benjamini-Hochberg multiple-test-corrected.

Of the 75 miRNAs connected to lung cancer prognostic genes (Figure 3), 15 were significantly associated with patient survival (Mantel Haenszel, logrank p <0.05), including miR-1 and miR-153 (Figure 4A and B and Additional file 13). When we assessed the association of any altered miRNAs with lung cancer patient survival in general (i.e. all lung tumor groups as a whole), we identified 76 miRNAs as significantly associated with LUAC patient survival (Mantel-Haenszel, logrank p <0.05), 22 of which were significant after correcting for multiple testing (B-H p <0.05) (Additional file 14). These included miRNAs previously associated with LUAC patient survival (miR-1247, let-7g, miR-146a, miR-126) [36–39] and recurrence (miR-200b) [40]. miR-187, which has been associated with brain metastasis in lung cancer patients [36] and which we observed as overexpressed in all smoking groups, was the most significant miRNA associated with survival when all LUAC were considered together (Benjamini-Hochberg corrected p =0.018, Additional file 15). Within individual smoking groups, 11 miRNAs were associated with patient survival in CS, 71 in FS, and 12 in NS (Table 2 and Additional file 14). Low expression of the CST-specific tumor suppressor miR-138 was also associated with poor survival in CS (p =0.009) (Figure 4C). High expression of EZH2, recently validated as a target of miR-138 in LUAC cells, was concordantly associated with poor patient survival (p =0.021, Figure 4D), further emphasizing the prognostic significance of miR-138.

**Table 2:** **Top 10 miRNAs significantly associated with lung adenocarcinoma patient survival in CS, FS, and NS**

miRNA	CS p-value	Status in tumors	miRNA	FS p-value	Status in tumors	miRNA	NS p-value	Status in tumors
hsa-mir-1287	0.0022	OE in ALL	hsa-mir-133a	0.0001	UE in ALL	hsa-mir-338	0.0006	UE in ALL
hsa-mir-138	0.0086	UE in CST	hsa-mir-429	0.0002	OE in ALL	hsa-let-7 g	0.0036	OE in ALL
hsa-mir-326	0.0131	OE in FST and NST	hsa-mir-642a	0.0005	OE in ALL	hsa-mir-184	0.0103	UE in CST and FST
hsa-mir-331	0.0146	OE in ALL	hsa-mir-153	0.0006	OE in ALL	hsa-mir-150	0.0143	OE in NST
hsa-mir-30d	0.0282	UE in ALL	hsa-mir-187	0.0010	OE in ALL	hsa-mir-139	0.0200	UE in ALL
hsa-mir-204	0.0291	UE in ALL	hsa-mir-21	0.0013	OE in ALL	hsa-mir-133b	0.0304	UE in ALL
hsa-mir-664	0.0331	OE in FST and NST	hsa-mir-26b	0.0018	OE in ALL	hsa-mir-664	0.0307	OE in FST and NST
hsa-mir-148a	0.0429	OE in ALL	hsa-mir-135b	0.0021	OE in ALL	hsa-mir-598	0.0326	UE in ALL
hsa-mir-195	0.0436	UE in CST	hsa-mir-3607	0.0022	OE in ALL	hsa-mir-10a	0.0342	UE in CST and FST
hsa-mir-1270	0.0462	OE in ALL	hsa-mir-99b	0.0027	OE in CST and FST	hsa-mir-92b	0.0351	OE in ALL

OE = overexpression; UE = underexpression.

Since miRNA survival associations could be confounded by association of the miRNAs with other clinical prognostic factors such as tumor stage, we determined the miRNAs most robustly associated with patient outcome using a multivariate survival analysis. We performed a Cox proportional hazards (COXPH) model on survival data for the 218 LUAC patients, considering miRNA expression, age, gender, stage, ethnicity and smoking status as clinical covariates potentially influencing patient outcome. In total, 103 miRNAs were detectably expressed in at least two-thirds of the 218 tumors. Of these 103 miRNAs assessed, 31 were associated with survival independently of tumor stage (COXPH p <0.1, Additional file 16). Interestingly, a corresponding multivariate analysis of variance (MANOVA) revealed that expression levels of all but one (miR-135b) of these 31 miRNAs were not related to tumor stage, suggesting they are indeed stage-independent lung cancer prognostic indicators (Additional file 16). Among the 31 miRNAs robustly associated with patient outcome were let-7g, miR-21, miR-1, and miR-138 (Figure 4), all of which have been previously implicated with lung cancer survival [39, 41–43]. Collectively, these analyses provide evidence of the biological importance and prognostic value of miRNA expression in the context of patient smoking history. Moreover, they provide additional rationale for smoking status-specific stratification of patients and evaluation of the roles of miRNA in LUAC biology in the context of smoking.

## Discussion

Cigarette smoke is associated with specific modifications to the genomic and epigenomic landscapes of airways and lung tissues [1, 44], affecting the transcriptional regulation of both genes and microRNAs (miRNAs) [12, 45–47]. Recent evidence suggests that histologically similar lung tumors harbor distinct molecular profiles based on smoking status, and that these alterations underlie observed clinical disparities between lung tumors in smokers and NS [3]. Since large LUAC cohorts with well annotated smoking histories have only recently become available, few studies have directly investigated smoking-associated molecular features of lung tumors on a genome-wide scale. To our knowledge, such a study designed to assess miRNA deregulation in the context of patient smoking history has yet to be performed. The LUAC dataset we have compiled is the largest lung miRNA-sequencing tumor cohort with well-defined smoking history and matched non-malignant lung tissue for every patient generated to date (n = 94 LUAC patients, 188 miRNA sequencing profiles).

Hierarchical clustering revealed that while smoking status and malignancy were associated with miRNA expression patterns, heterogeneity amongst CS and FS is present (Figure 1). It is likely that, in addition to other non-miRNA molecular alterations, inter-individual genetic variants involved in the biological response to smoking may underlie this observed heterogeneity [48–50]. Analogous to observations for protein-coding genes [23, 24], we identified miRNAs differentially expressed between CSN and NSN and either irreversibly (miR-107, -378c, -142 and -34c) or reversibly (miR-3648 and miR-3687) expressed in FSN (Additional file 4). It is plausible that altered expression of miRNAs in CSN and FSN tissues may be an early event related to smoking-associated tumorigenesis, although without interrogation of lung tissues from CS, FS and NS individuals *without* lung cancer it is difficult to distinguish smoking-induced alterations from those that are related to lung cancer itself. Investigation into the biological contribution of these "irreversible" miRNAs is warranted to determine whether they do indeed contribute to lung tumorigenesis.

The majority of frequently disrupted miRNAs in LUAC relative to non-malignant lung tissues were commonly disrupted across all tumor groups, indicating that despite different smoking histories, common biological mechanisms largely underlie LAUC tumorigenesis. However, we found smoking status-specific miRNAs that were predicted to target unique mRNA transcripts, suggesting that miRNAs may also contribute to mechanisms of LUAC tumorigenesis specific to distinct smoking environments. For example, gene targets unique to miRNAs disrupted in CST, FST or NST could be indicative of the importance of these miRNAs to LUAC biology specifically in these groups. Conversely, genes heavily targeted by different miRNAs distinctly altered in CST, FST or NST may indicate the importance of these genes to LUAC biology, irrespective of smoking status.

We acknowledge that the lack of validation of the smoking status-specific miRNA deregulation we observed in external datasets is a limitation of this study. Due to i) small sample sizes, ii) lack of smoking history annotation, iii) lack of patient-matched non-malignant tissue profiles for defining miRNAs as over- or under-expressed in individual tumors, and iv) use of miRNA expression arrays for profiling which drastically reduces the number of measureable miRNAs, existing external miRNA LUAC expression datasets are not directly appropriate for validation. The TCGA, which represents the largest public repository for LUAC miRNA expression data generated by sequencing, contains a small number of NST (n = 16) and few patient-matched LUAC tumor and non-malignant profiles. However, the large number of miRNA sequencing expression profiles with annotated smoking histories from the TCGA were integral in enabling smoking status-specific survival analyses in LUAC patients. We note that our findings may still be limited by small sample size, especially the CS and NS groups. Another potential caveat to our study is the large proportion of never smoker patients of Asian ethnicity. Thus, we stress that validation of our findings in external cohorts is necessary. As the cost and sample requirements associated with accruing such data continue to decline, and the importance of considering smoking status in lung cancer patient management is increasingly appreciated, the availability of large, annotated lung cancer datasets will undoubtedly grow, enabling validation of our findings.

A large number of lung cancer prognostic genes were identified as predicted targets for both commonly disrupted and smoking status-specific miRNAs [21]. CST-specific miR-372 targeted the most lung cancer prognostic genes and is a known oncogenic miRNA associated with poor outcome and aggressive disease in multiple cancers; in lung cancer, it is a strong candidate for use as an early detection sputum-based biomarker [26, 27, 51–53]. The numerous mRNA targets of miR-372 were recently described in a LUAC comparative proteomic analysis, further alluding to the extensive pro-tumorigenic role of this miRNA in lung cancer [54]. However, despite its frequent overexpression in CST (26%), the low variability in miR-372 expression levels across CST prevented us from statistically assessing the association between miR-372 expression levels and lung cancer patient survival in our study.

The degree of overlap between miRNAs and prognostic mRNA targets was particularly high for miRNAs specifically disrupted in CST or FST. We suspect this is due to the fact that the lung cancer prognostic signatures we analyzed were based on typical lung cancer patient cohorts which contain small numbers of NST. Our findings underscore the potential clinical significance of miRNAs frequently altered in LUAC and illustrate that disruption of a small number of miRNAs can potentially introduce an enormous amount of biological complexity.

In contrast to previous studies where the association of miRNA expression and survival was conducted in relation to NSCLC histological subtypes, mutational status, or tumor stage [36, 55–58], we conducted an analysis of miRNA expression associated with lung cancer patient survival in relation to smoking status. We identified numerous miRNAs significantly associated with patient prognosis in different smoking status groups or in LUAC in general (Table 2 and Additional file 14). Significantly, several of these miRNAs were associated with survival independent of tumor stage based on a Cox proportional hazards multivariate model of patient outcome (Additional file 16). Of interest, miR-195, miR-138 and miR-150, which demonstrated recurrent, aberrant expression in a specific smoking status group, were also significantly associated with survival in that same group.

Low expression levels of miR-195 are associated with poor patient prognosis in glioblastoma and colon cancer [59, 60], consistent with the prognostic association we identified for this miRNA in LUAC of CST. In the context of cigarette smoke and non-malignant lung disease, miR-138 may have a role in hypoxic pulmonary vascular remodelling and pulmonary arterial hypertension through its role in the negative regulation of pulmonary artery smooth muscle cell apoptosis [61]. A recent study by Zhang *et al*. not only validated an anti-tumorigenic role for miR-138, but also demonstrated that this action occurred through targeted inhibition of EZH2 by miR-138 [62]. This study provides independent validation of our target prediction methods and provides further biological evidence of the importance of miR-138 to lung cancer. MiR-150 is a candidate oncogenic miRNA, although its role in lung cancer is ambiguous [63–65]. Sun *et al.* report that expression of miR-150 is significantly downregulated in tumor tissues and embryonic lung tissues compared to normal lung tissues, although preferentially in tumors from smokers [63]. Two additional studies identified upregulation of miR-150 in lung tumors, demonstrating a link between lung cancer cell proliferation and miR-150 through targeted inhibition of *TP53* [64, 65]. We observed frequent (40%) overexpression of miR-150 specifically in NST, and found its overexpression was associated with better prognosis in NS LUAC patients. Thus, the mechanisms contributing to the prognostic significance of miR-150 in LUAC may be related to the biology underlying lung tumorigenesis in NS.

It is worthy to note that pending additional external cohort validation of our findings, the miRNAs we have identified may serve as clinically relevant biomarkers or therapeutic targets, as has been demonstrated for numerous miRNAs deregulated in cancer [66–68]. Given the stability of miRNAs in biological samples such as blood and the relative ease with which miRNAs can be extracted from clinical specimens, miRNAs are ideal molecules to investigate as biomarkers. For instance, if detectable in surrogate tissues the candidate miRNAs we have identified as overexpressed could be evaluated for their potential to detect malignancy in lung samples such as bronchial brushings or bronchoalveolar lavage or even in blood. Moreover, it has been proposed that miRNAs may represent therapeutic intervention points [69, 70]. For example, miRNA sponges may be used to "mop up" overexpressed miRNAs to prevent them from inhibiting their respective mRNA targets, and understanding the biological pathways miRNA deregulation affects could also shed light on new therapeutic strategies. Since the clinical relevance of the candidates we have revealed could be promising, future studies to evaluate whether these miRNAs have clinical utility are warranted.

## Conclusions

In conclusion, our study suggests that patterns of miRNA deregulation promote smoking-specific LUAC biology, but also highlights shared biology underlying LUAC tumorigenesis across all smoking and non-smoking groups. Given the relatively small sample size and the associated potential confounding factors including ethnicity, which was not matched across the smoking groups we studied, external validation of our findings in prospective cohorts is warranted. Nevertheless, our results reaffirm the extent to which miRNAs can contribute to the molecular complexity of cancer genomes and suggest that miRNA disruption may contribute to the differential development and distinct clinical features observed in CS, FS, and NS lung cancer patients.

## Electronic supplementary material

Additional file 1: **miRNA differentially expressed between clusters identified in tumor and non-malignant tissues.** (XLSX 18 KB)

Additional file 2: **MANOVA results for miRNA expression profile clustering.** (XLS 20 KB)

Additional file 3: **miRNAs differentially expressed in non-malignant lung tissue of patients with lung adenocarcinoma.** (XLSX 12 KB)

Additional file 4: **miRNA expression in non-malignant lung tissues can be irreversibly altered in former smokers.** (XLSX 13 KB)

Additional file 5: **miRNA differentially altered between tumor and normal pairs from CS, FS and NS.** (XLS 61 KB)

Additional file 6: **Venn diagram illustrating differentially expressed miRNAs in lung tumors relative to matched non-malignant tissues from CS, FS, and NS.** (PDF 249 KB)

Additional file 7: **miRNA with specific deregulation in CS, FS, and NS lung adenocarcinoma.** (XLSX 13 KB)

Additional file 8: **Comparison of RPKM values and miRNA detection in the TCGA and BCCA datasets.** (PDF 263 KB)

Additional file 9: **Four miRNA validated as specifically disrupted in one smoking group.** (PDF 264 KB)

Additional file 10: **Canonical pathways differentially and commonly enriched for biologically validated target genes of miRNA specifically deregulated in one smoking group.** (PDF 265 KB)

Additional file 11: **Pathways significantly enriched for biologically validated target genes of miRNA specifically disrupted in CS, FS, and NS lung AC.** (XLS 63 KB)

Additional file 12: **miRNA and predicted mRNA targets comprising Figure** 3 **.** (XLS 78 KB)

Additional file 13: **miRNA associated with lung AC patient survival that target lung cancer prognostic genes.** (XLS 32 KB)

Additional file 14: **miRNA identified as having significant associations between miRNA expression and lung AC patient survival.** (XLS 45 KB)

Additional file 15: **miR-187 is the most significant miRNA associated with patient survival.** (PDF 240 KB)

Additional file 16: **Multivariate analysis of miRNA expression and patient survival.** (XLSX 41 KB)

## Abbreviations

LUAC: Adenocarcinoma
B-H: Benjamini–Hochberg
CS: Current smoker
CSN: Current smoker non-malignant
CST: Current smoker tumor
FC: Fold change
FS: Former smoker
FSN: Former smoker non-malignant
FST: Former smoker tumor
miR: Hsa-mir
miRNA: microRNA
NAViGaTOR: Network analysis, visualization, & graphing Toronto
ncRNA: Non-coding RNA
NS: Never smoker
NSN: Never smoker non-malignant
NST: Never smoker tumor
RPKM: Reads per kilo base per million
TCGA: The cancer genome atlas.
