# Supplementary material for: Smoking status impacts microRNA mediated prognosis and lung adenocarcinoma biology
Source: BMC Cancer. 2014 Oct 24;14:778. doi: 10.1186/1471-2407-14-778 (PMC4216369; doi:10.1186/1471-2407-14-778)
Supplement: Supplementary file 6 — Additional file 6: Venn diagram illustrating differentially expressed miRNAs in lung tumors relative to matched non-malignant tissues from CS, FS, and NS. (PDF 249 KB) [file 12885_2014_4957_MOESM6_ESM.pdf]

Additional File 6

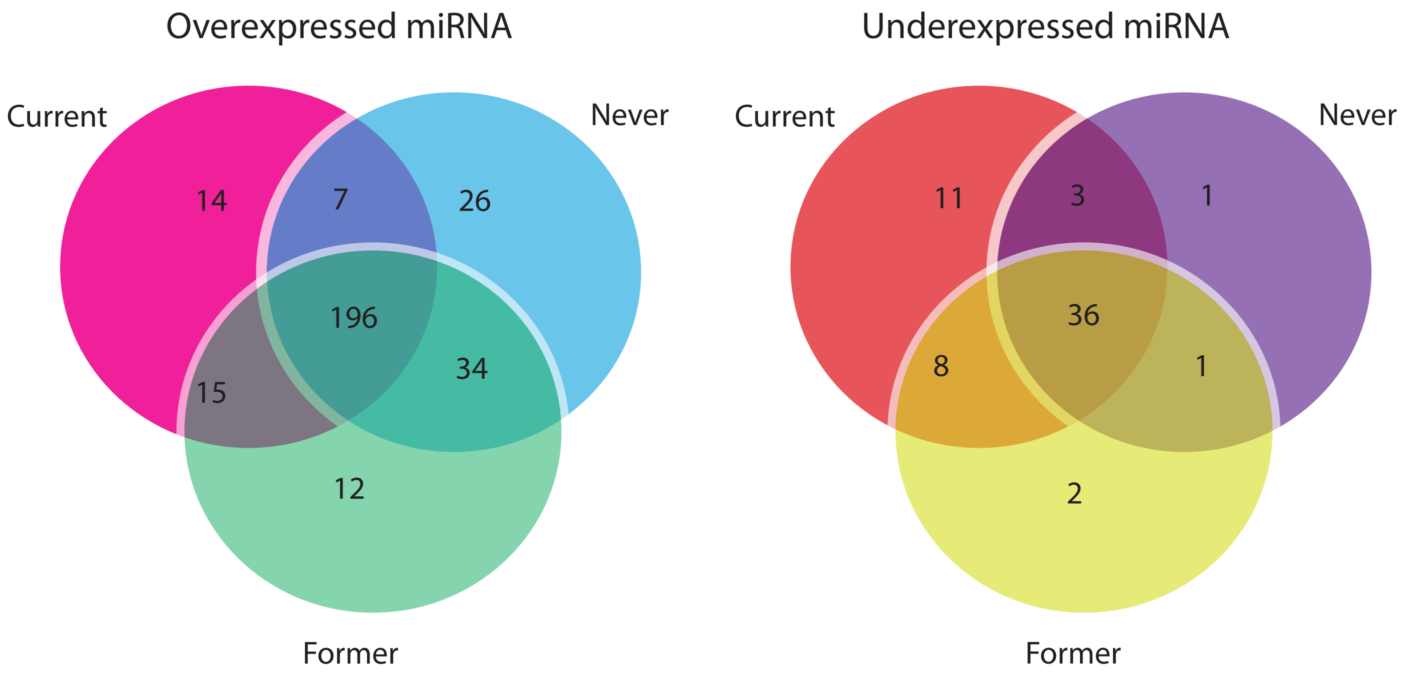

Additional File 6. Venn diagram illustrating differentially expressed miRNAs in lung tumors relative to matched non-malignant tissues from CS, FS, and NS. miRNAs recurrently (>25%) disrupted and significantly, differentially expressed between paired, tumor and patient matched non-malignant lung tissues were assessed to determine the overlap in disruption between the groups. Overexpressed miRNAs are depicted in (A) and underexpressed miRNAs in (B). The majority of miRNAs differentially expressed between tumor and non-malignant tissues were overexpressed and most of the miRNAs identified were deregulated in all three smoking groups.
