# Supplementary material for: Smoking status impacts microRNA mediated prognosis and lung adenocarcinoma biology
Source: BMC Cancer. 2014 Oct 24;14:778. doi: 10.1186/1471-2407-14-778 (PMC4216369; doi:10.1186/1471-2407-14-778)
Supplement: Supplementary file 8 — Additional file 8: Comparison of RPKM values and miRNA detection in the TCGA and BCCA datasets. (PDF 263 KB) [file 12885_2014_4957_MOESM8_ESM.pdf]

Additional File 8

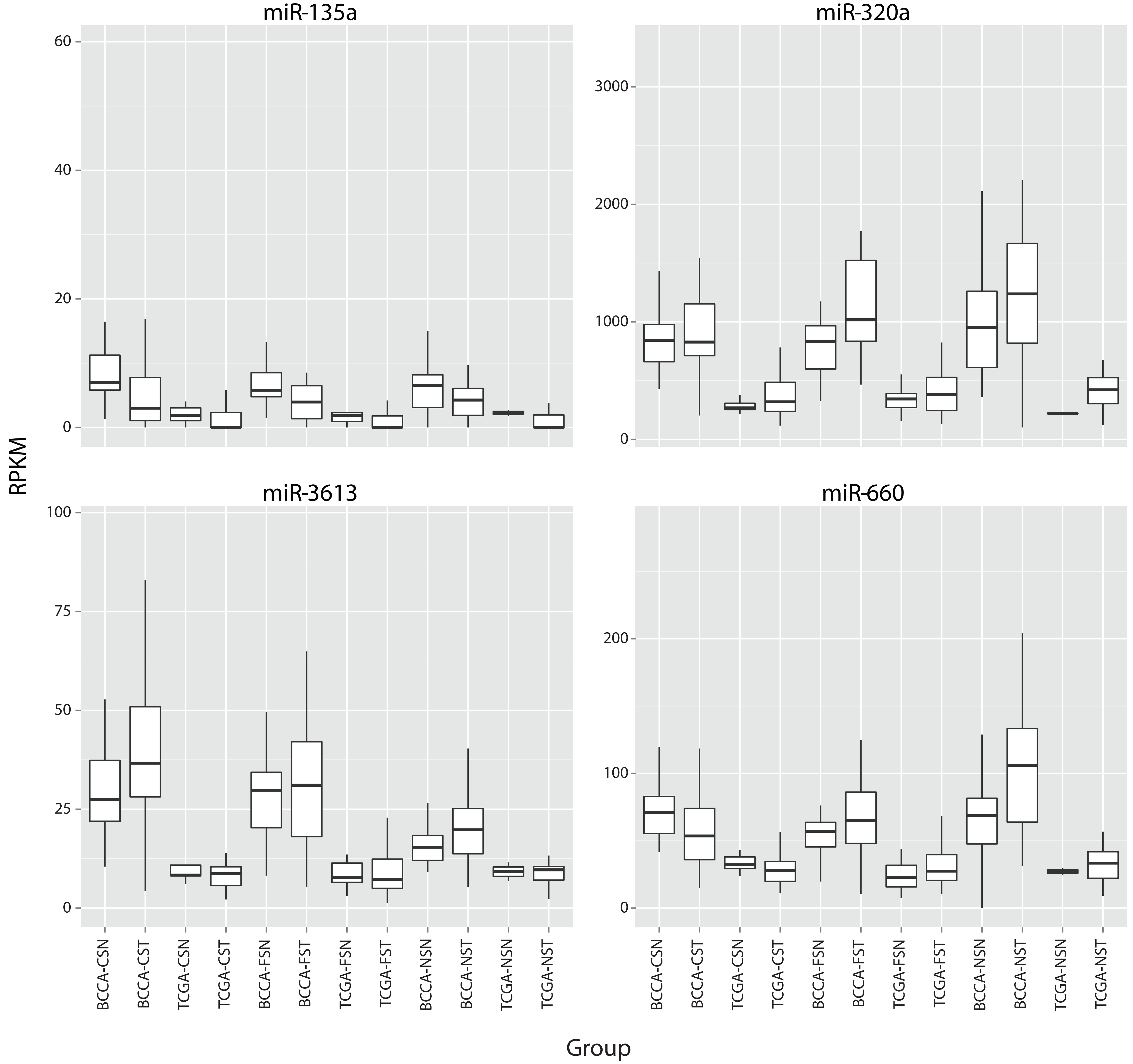

Additional File 8. Comparison of RPKM values and miRNA detection in the TCGA and BCCA datasets. Normalized miRNA expression values (RPKM) are plotted for CS, FS, and NS in our BCCA cohort and the TCGA dataset. The four miRNA illustrated demonstrate the difference in miRNA detection between tumour and non-malignant samples between the two datasets; miRNA in the TCGA data have lower expression values. These features of the TCGA data make validation of differentially disrupted miRNA difficult. The low validation rate we observed (4/45 assessable miRNA) likely reflects the compressed nature of the TCGA expression data. The box contains the 25th and 75th percentiles of expression data and the horizontal line indicates the median of the data. Whiskers extending from the box indicate the highest and lowest values within 1.5 times the interquartile range.
