# Supplementary material for: Smoking status impacts microRNA mediated prognosis and lung adenocarcinoma biology
Source: BMC Cancer. 2014 Oct 24;14:778. doi: 10.1186/1471-2407-14-778 (PMC4216369; doi:10.1186/1471-2407-14-778)
Supplement: Supplementary file 9 — Additional file 9: Four miRNA validated as specifically disrupted in one smoking group. (PDF 264 KB) [file 12885_2014_4957_MOESM9_ESM.pdf]

Additional File 9

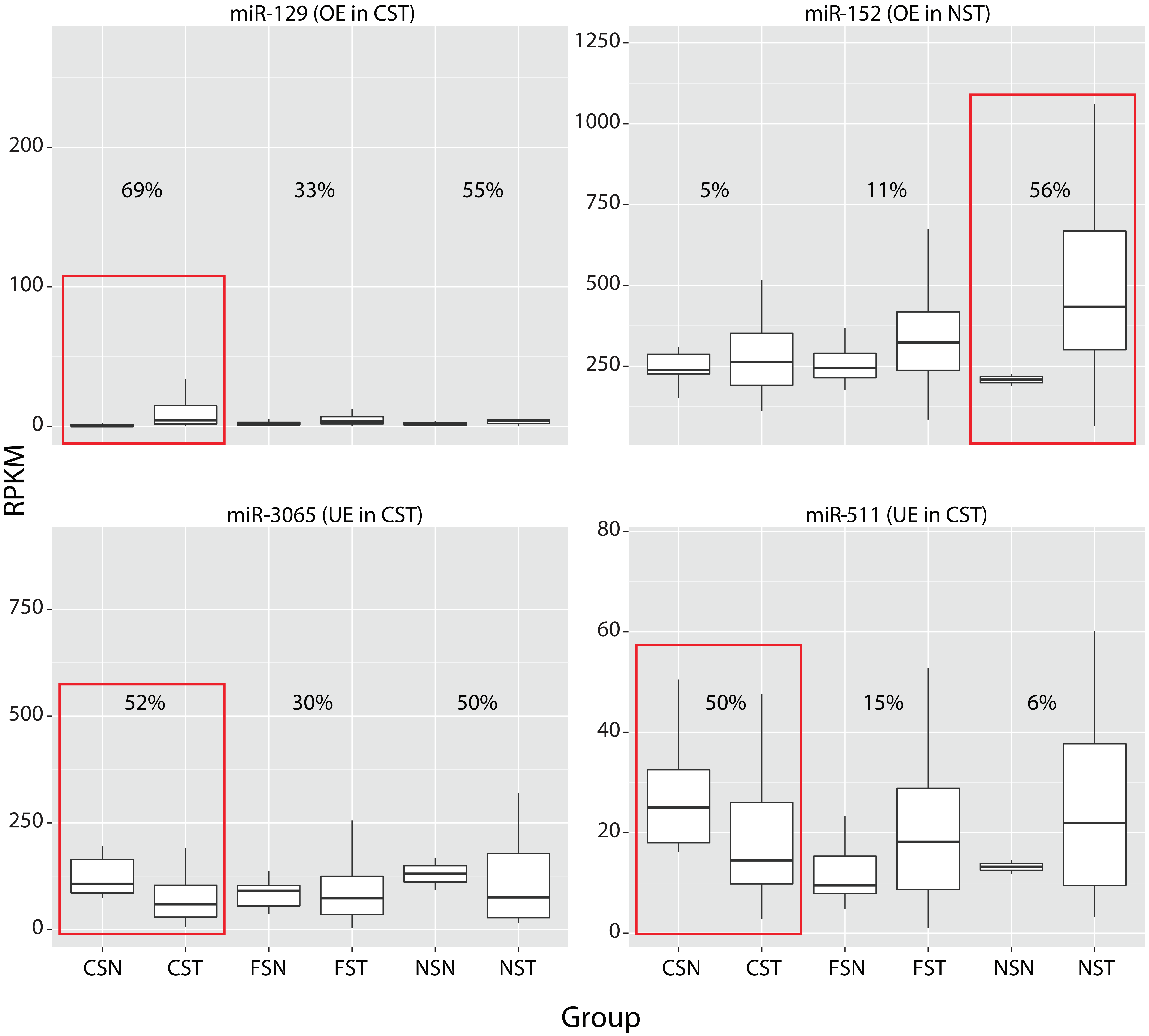

Additional File 9. Four miRNA validated as specifically disrupted in one smoking group. Boxplots illustrate expression values of the four miRNA we validated as disrupted in a smoking specific manner in the TCGA cohort. Red boxes indicate the group in which miRNA disruption (over- or underexpression) occurs. Frequencies of miRNA disruption in tumour relative to non-malignant samples are indicated for each smoking group. miRNAs were considered validated if they exhibited a significant difference in alteration frequency between smoking groups (Fisher's exact test, $p < 0.05$) and a minimum 15% frequency difference between smoking groups concordant with our findings. CST, FST, NST = current, former and never smoker tumors, respectively. CSN, FSN, NSN = current, former and never smoker non-malignant, respectively. OE = overexpression, UE = underexpression.
