# Supplementary material for: Smoking status impacts microRNA mediated prognosis and lung adenocarcinoma biology
Source: BMC Cancer. 2014 Oct 24;14:778. doi: 10.1186/1471-2407-14-778 (PMC4216369; doi:10.1186/1471-2407-14-778)
Supplement: Supplementary file 10 — Additional file 10: Canonical pathways differentially and commonly enriched for biologically validated target genes of miRNA specifically deregulated in one smoking group. (PDF 265 KB) [file 12885_2014_4957_MOESM10_ESM.pdf]

Additional File 10

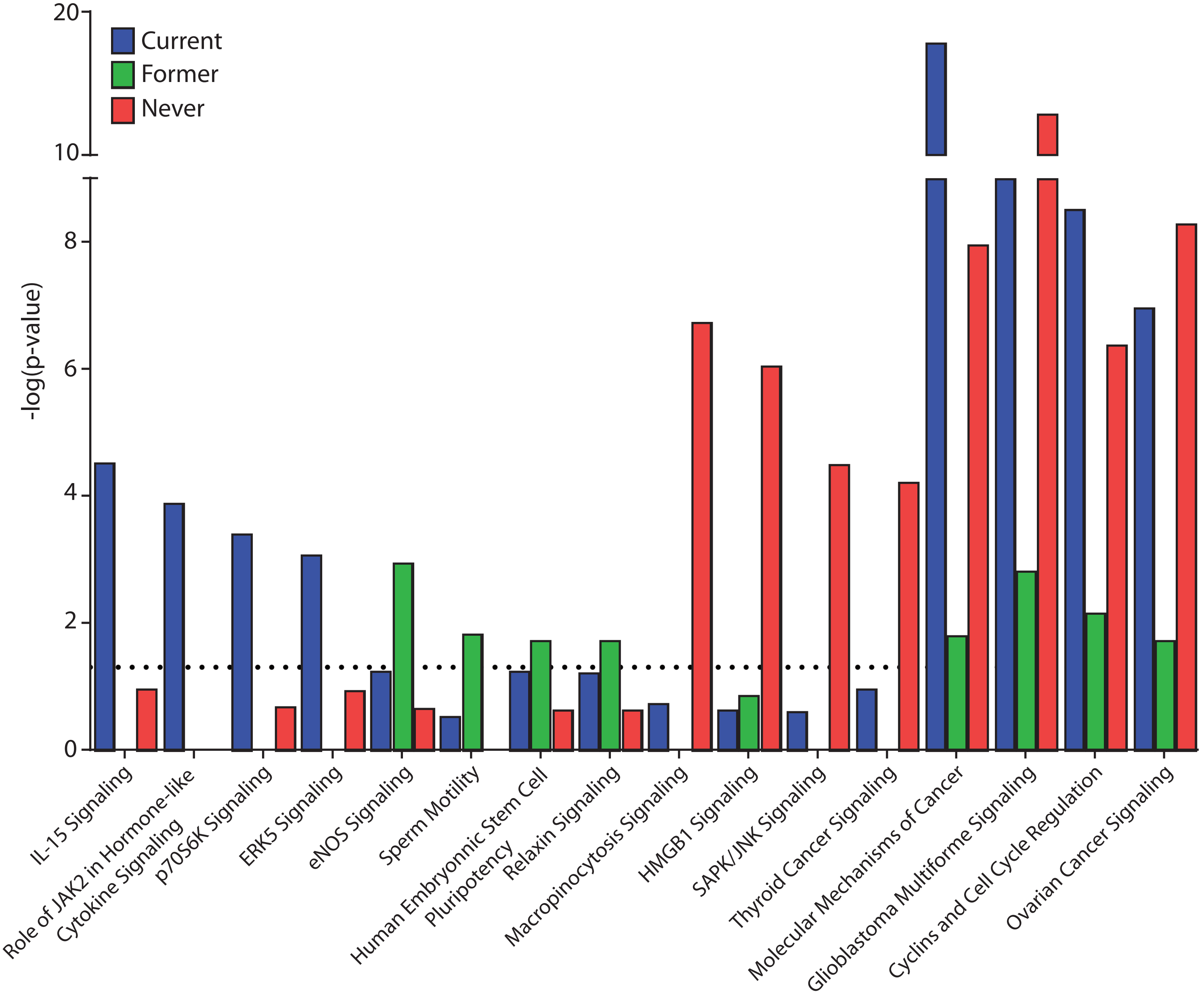

Additional File 10. Canonical pathways differentially and commonly enriched for biologically validated target genes of miRNA specifically deregulated in one smoking group. Pathway analysis was performed on biologically validated gene targets (based on miRTarBase v3.5) of miRNAs. Analyses were conducted separately for targets of miRNA specific to CS, FS, and NS. All annotated canonical pathways significantly enriched across all groups or in a smoking-status specific manner are listed in Additional File 11. Select pathways are illustrated, including examples of those specific to CS, FS, and NS, and common across all smoking groups. The dotted horizontal line indicates the threshold for significant pathway enrichment (Fisher's Exact test $p < 0.05$).
