# Supplementary material for: Smoking status impacts microRNA mediated prognosis and lung adenocarcinoma biology
Source: BMC Cancer. 2014 Oct 24;14:778. doi: 10.1186/1471-2407-14-778 (PMC4216369; doi:10.1186/1471-2407-14-778)
Supplement: Supplementary file 15 — Additional file 15: miR-187 is the most significant miRNA associated with patient survival. (PDF 240 KB) [file 12885_2014_4957_MOESM15_ESM.pdf]

Additional File 15

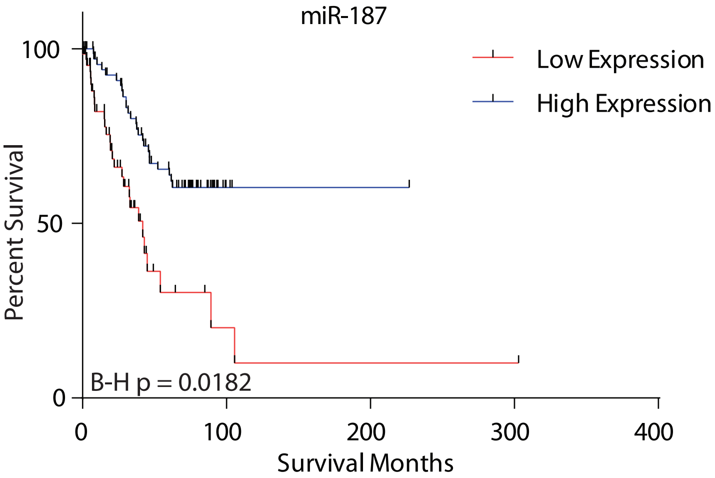

Additional File 15. miR-187 is the most significant miRNA associated with patient survival. Associations between miRNA expression and LUAC patient survival were assessed using a logrank, Mantel-Haenszel test. Considering all patient samples combined, regardless of smoking histories, miR-187 was the disrupted miRNA most significantly associated with patient survival after multiple testing correction.
